# Determination of antimicrobial susceptibility patterns in *Staphylococcus aureus* strains recovered from patients at two main health facilities in Kabul, Afghanistan

**DOI:** 10.1186/s12879-017-2844-4

**Published:** 2017-11-29

**Authors:** Haji Mohammad Naimi, Hamidullah Rasekh, Ahmad Zia Noori, Mohammad Aman Bahaduri

**Affiliations:** Department of Microbiology, Faculty of Pharmacy, Kabul University, Jamal Meena street, Kabul, Afghanistan

**Keywords:** MRSA, Kabul health facilities, Antimicrobial susceptibility

## Abstract

**Background:**

*Staphylococcus aureus (S. aureus)* is a major pathogen implicated in skin and soft tissue infections, abscess in deep organs, toxin mediated diseases, respiratory tract infections, urinary tract infections, post-surgical wound infections, meningitis and many other diseases. Irresponsible and over use of antibiotics has led to an increased presence of multidrug resistant organisms and especially methicillin resistant *Staphylococcus aureus* (MRSA) as a major public health concern in Afghanistan. As a result, there are many infections with many of them undiagnosed or improperly diagnosed. We aimed to establish a baseline of knowledge regarding the prevalence of MRSA in Kabul, Afghanistan, as well as *S. aureus* antimicrobial susceptibility to current available antimicrobials, while also determining those most effective to treat *S. aureus* infections.

**Methods:**

Samples were collected from patients at two main Health facilities in Kabul between September 2016 and February 2017. Antibiotic susceptibility profiles were determined by the disc diffusion method and studied using standard CLSI protocols.

**Results:**

Out of 105 strains of *S. aureus* isolated from pus, urine, tracheal secretions, and blood, almost half (46; 43.8%) were methicillin-sensitive *Staphylococcus aureus* (MSSA) while 59 (56.2%) were Methicillin-resistant *Staphylococcus aureus* (MRSA). All strains were susceptible to vancomycin. In total, 100 (95.2%) strains were susceptible to rifampicin, 96 (91.4%) susceptible to clindamycin, 94 (89.5%) susceptible to imipenem, 83 (79.0%) susceptible to gentamicin, 81(77.1%) susceptible to doxycycline, 77 (77.1%) susceptible to amoxicillin + clavulanic acid, 78 (74.3%) susceptible to cefazolin, 71 (67.6%) susceptible to tobramycin, 68 (64.8%) susceptible to chloramphenicol, 60 (57.1%) were susceptible to trimethoprim-sulfamethoxazole, 47 (44.8%) susceptible to ciprofloxacin, 38 (36.2%) susceptible to azithromycin and erythromycin, 37 (35.2%) susceptible to ceftriaxone and 11 (10.5%) were susceptible to cefixim. Almost all (104; 99.05%) were resistant to penicillin G and only 1 (0.95%) was intermediate to penicillin G. Interestingly, 74.6% of MRSA strains were azithromycin resistant with 8.5% of them clindamycin resistant. Ninety-six (91.4%) of the isolates were multi-drug resistant.

**Conclusions:**

There was a high rate of Methicillin resistance (56.2%) among *S. aureus* strains in the samples collected and most (91.4%) were multidrug resistant. The most effective antibiotics to treat *Staph* infections were vancomycin, rifampicin, imipenem, clindamycin, amoxicillin-clavulanic acid, cefazolin, gentamicin and doxycycline. The least effective were azithromycin, ceftriaxone, cefixim and penicillin. We recommend that, where possible, in every case of *S. aureus* infection in Kabul, Afghanistan, Antibiotic susceptibility testing (AST) should be performed and responsible use of antibiotics should be considered.

## Background

*Staphylococcus aureus* is a major pathogen implicated in skin and soft tissue infections [1]. Multidrug resistance in *Staphylococci* is an increasing problem in clinical practice especially methicillin-resistant *S. aureus* (MRSA) strains. These strains are resistant to most of the antimicrobial agents, and isolates with reduced susceptibility and resistance to vancomycin, which is the last drug for the treatment of MRSA infections [2]. These multidrug resistant strains may cause severe infections with a high rate of mortality. In vitro susceptibilities of MRSA strains, especially those from community-acquired infections, to clindamycin, macrolides, quinolones, tetracyclines, and trimethoprim-sulfamethoxazole have frequently been reported [3, 4]. Strains of MRSA, which had been largely confined to hospitals and long-term care facilities, are emerging elsewhere in the community. The changing epidemiology of MRSA bears striking similarity to the emergence of penicillinase-mediated resistance in *S. aureus* decades ago [5]. One of the best choices of treatment of MRSA is to treat with clindamycin and fluoroquinolones such as ciprofloxacin, but recent studies showed that susceptibility of this microorganism is also decreasing to clindamycin and fluoroquinolones [6, 7].

In a study in southern districts of Tamilnadu, India [8], the prevalence of MRSA strains isolated from clinical and carrier samples were 37.9%. Almost all clinical MRSA strains (99.6%) were resistant to penicillin, 93.6% to ampicillin, and 63.2% towards gentamicin, co-trimoxazole, cephalexin, erythromycin, and cefotaxime. All MRSA strains (100%) of carrier screening samples had resistance to penicillin and 71.8% and 35.9% respectively were resistant to ampicillin and co-trimoxazole. However, all strains of clinical and carrier subjects were sensitive to vancomycin. In this study, it was concluded that the determination of prevalence and antibiotic susceptibility patterns of MRSA would help the treating clinicians for first line treatment in referral hospitals.

A study in a hospital in Turkey aimed to determine the susceptibility patterns of *Staphylococcus aureus* strains to various antimicrobials, 50.2% were resistant to methicillin. All strains were susceptible to vancomycin, teicoplanin, quinupristin/dalfopristin, and linezolid. It was found that 53.4% MRSA strains were erythromycin resistant, and 39.6% showed constitutive clindamycin resistance. In this study they identified the high rate of methicillin resistance among *S. aureus* strains in their hospital [9].

In Afghanistan the widespread use of antibiotics has led to increase in the number of multidrug resistant organisms including MRSA [10, 11]. A study in Afghanistan, showed that there is a significant amount of overuse and abuse of antimicrobials in primary health care clinics that may lead to problem of antimicrobial resistance [12]. Another study at a US military hospital in Bagram Airbase in Afghanistan, found that Afghan patients often carry multidrug-resistant (MDR) bacteria compared to US citizens treated in this hospital. Their findings suggested the need for effective infection control measures at deployed hospitals where both soldiers and local patients are treated [13].

Indeed, some strains have become resistant to practically all of the commonly available antibiotics in Afghanistan. That is why the physicians mostly prescribe new antibiotics in order to get positive results without knowing the susceptibility patterns of causative bacterial agents [11]. There is no study regarding the prevalence of MRSA which is a multidrug resistant bacteria and its susceptibility patterns to most common antibiotics in Afghanistan, which causes severe infections with a higher mortality rate both community and hospital acquired infections. The aim of this study is to assess the prevalence of MRSA, as well as determining antimicrobial susceptibility patterns of *S. aureus* strains to common antibiotics available in Kabul, Afghanistan. The results of this study would help physicians in Kabul to know the prevalence of MRSA and to help them change their treatment protocols, and to know the importance of bacteriological culture and antibiotic susceptibility testing (AST). It would also be helpful for the Ministry of Health of Afghanistan to pay more attention to diagnostic labs and the role of bacteriological culture and AST to provide better treatment outcomes and responsible use of antibiotics. The findings would also emphasize the importance of local surveillance in generating relevant local resistance data that can guide empiric therapy.

## Methods

This longitudinal study was conducted in the Microbiology Laboratory of the Faculty of Pharmacy of Kabul University between September 2106 and February 2017. Presumptive isolates from various clinical samples were brought from two main health facilities of Kabul, to the microbiology lab of the Faculty of Pharmacy. All of the isolates were collected from clinical specimens obtained from hospitalized patients. The standard microbiological procedures were conducted with minimum delay for culture, confirmatory tests and AST. We selected two main health facilities in Kabul, because they have standard microbiology labs and perform most of the bacteriological cultures and identification in Kabul. Confirmatory tests were carried out for diagnosis of *S. aureus strains*, by inoculating presumptive isolates onto Blood agar base medium (Oxoid, England) to which 5% sheep blood was added. All cultured media were incubated at 37 °C for 18–24 h under aerobic condition. The suspected isolated colonies were subjected to Gram’s staining, Catalase test, Coagulase test, and Mannitol fermentation on Mannitol Salt agar (Oxoid, England) [14]. Confirmed *S. aureus* isolates were subjected to AST by Kirby Bauer disc diffusion method as per Clinical Laboratory Standards Institute (CLSI) guidelines [15] on Muller Hinton agar (Oxoid, England) for 20 antimicrobials such as: penicillin G (P,1unit), amoxicillin-clavulanic acid (AMC, 30μg), oxacillin (OX, 1μg), azithromycin (ATH, 15μg), erythromycin (E, 15μg), cefazolin (CZ, 30μg), ceftazidime (CAZ, 30μg), cefoxitin (FOX, 30μg), cefixim (CFM, 5μg), ceftriaxone (CRO, 30μg), ciprofloxacin (CIP, 5μg), trimethoprim-sulfamethoxazole (SXT, 1,25/23,75μg), gentamicin (CN, 10μg), tobramycin (TOB, 10μg), doxycycline (DO, 30μg), imipenem (IMI, 10μg), clindamycin (CD, 2μg), vancomycin (VA, 30μg), chloramphenicol (C, 10μg) and rifampicin (RP, 5μg).

The growth suspension for AST was prepared in 5 ml Normal saline solution and the turbidity was adjusted to match that of 0.5 McFarland standards to obtain approximately the organism number of 1 × 10^6^ colony forming units (CFU) per ml. Antibiotic discs were placed after 15 min of inoculation to Muller Hinton agar seeded with each isolate and were incubated for 18–24 h at 35–37 °C. The diameter of the zone of inhibition around the disc was measured using sliding metal caliper. For accuracy, during the antibiotic screens, three independent replicates were performed. The susceptibility of all isolates were determined against different classes of antibiotics as follows:

For detection of MRSA we applied two definitions: [1] inhibition zone less than or equal to 23 mm on Mueller Hinton Agar (MHA) with 30 μg cefoxitin disc seeded with growth suspension of *S. aureus* isolates adjusted to 0.5 McFarland standards at 37 °C for 18-24 h [16]; [2] inhibition zone on MHA containing 2% NaCl with 1μg oxacillin disc less than or equal to 10 mm seeded with growth suspension of *S. aureus* isolates adjusted to 0.5 McFarland standards at 30 °C for 18-24 h [17].

For detection of Multi Drug Resistance, we used the definition of *Magiorakos* et al. [18] as non-susceptibility to at least one agent in three or more antimicrobial categories.

### Statistical analysis and quality assurance

The reliability of the study findings was guaranteed by implementing quality control measures throughout the whole processes of laboratory work. We used two strains of *S. aureus* as control. *S. aureus* ATCC 29213 a *mecA* negative strain, and *S. aureus* ATCC 43300 a *mecA* positive strain; both confirmed with standard PCR as reference methicillin-sensitive *S. aureus* (MSSA) and MRSA strains respectively using the DNA amplification instrument Mastercycler gradient (Eppendorf, Germany).

The statistical analysis was done using SPSS version 19. Binary logistic regression was used to determine the association between *S. aureus* infection, gender and age. Multivariate logistic regressions were used to control confounding factors. A *P-value* less than 0.05 was considered as statistically significant.

## Results

Of 105 strains of *S. aureus* isolated from various types of pus, urine, tracheal secretions and blood, 46 (43.8%) were MSSA while 59 (56.2%) were MRSA. All strains (105; 100%) were susceptible to vancomycin. Almost all (104; 99.05%) were resistant to penicillin G and only 1 (0.95%) was intermediate to penicillin, for further information please refer to Table 1.

**Table 1:** Antimicrobial susceptibility patterns of *S. aureus* strains to different antimicrobial agents

Classes of ATB	Antibiotics	Sensitive N (%)	Intermediate N (%)	Resistant N (%)
Penicillines	Penicillin G		1 (1.0)	104 (99.0)
	Amoxicillin + Clavulanic acid	81 (77.1)		24 (22.9)
	Oxacillin	49 (46.7)		56 (53.3)
Macrolides	Erythromycin	38 (36.2)	4 (3.8)	63 (60.0)
	Azithromycin	38 (36.2)	4 (3.8)	63 (60.0)
2nd and 3rd generation of Cephalosporins	Cefazolin	78 (74.3)	10 (9.5)	17 (16.2)
	Cefixim	11 (10.5)	5 (4.8)	89 (84.8)
	Cefoxitin	49 (46.7)		56 (53.3)
	Ceftriaxone	37 (35.2)	39 (37.1)	29 (27.6)
	Ceftazidime	6 (5.7)	12 (11.4)	87 (82.9)
Quinolones	Ciprofloxacin	47 (44.8)	5 (4.8)	53 (50.5)
Sulfonamides	Cotri-moxazole	60 (57.1)	9 (8.6)	36 (34.3)
Aminoglycosides	Gentamicin	83 (79.0)	5 (4.8)	17 (16.2)
	Tobramycin	71 (67.6)		34 (32.4)
Tetracycline	Doxycycline	81 (77.1)	10 (9.5)	14 (13.3)
Carbapenems	Imipenem	94 (89.5)	1 (1.0)	10 (9.5)
Lincosamides	Clindamycin	96 (91.4)	4 (3.8)	5 (4.8)
Polypeptides	Vancomycin	105 (100.0)		
Divers	Rifampicin	100 (95.2)		5 (4.8)
	Chloramphenicol	68 (64.8)	30 (28.6)	7 (6.7)

We did not find any strain of MSSA to be resistant to clindamycin and only 6.5% were intermediate to clindamycin, while 8.5% of MRSA strains were resistant to clindamycin. Susceptibility to azithromycin was low in both MSSA (52.2%) and MRSA (23.7%). MSSA vs MRSA isolates showed a higher susceptibility to amoxicillin + clavulanic acid, 2nd and 3rd generation of cephalosporins, aminoglycosides, imipenem, ciprofloxacin, rifampicin, and co-trimoxazole, for further information please refer to Table 2.

**Table 2:** Comparative susceptibility of MRSA *and* MSSA strains to different antimicrobial agents

Classes of ATB	Antibiotics	MSSA (%)	MRSA (%)	*P-value*
Penicillines	Penicillin G	0	0	
	Amoxicillin + Clavulanic acid	97.8	61	**0.0001**
Macrolides	Erythromycin	52.2	23.7	**0.002**
	Azithromycin	52.2	23.7	**0.002**
2nd and 3rd generation of Cephalosporins	Cefazolin	97.8	55.9	**0.0001**
	Cefixim	19.6	3.4	**0.001**
	Ceftriaxone	71.7	6.8	**0.0001**
	Ceftazidime	8.7	3.4	**0.005**
Quinolones	Ciprofloxacin	60.9	32.2	**0.0001**
Sulfonamides	Cotri-moxazole	69.6	47.5	**0.004**
Aminoglycosides	Gentamicin	95.1	66.7	**0.001**
	Tobramycin	82.6	55.9	**0.004**
Tetracyclines	Doxycycline	71.7	81.4	0.478
Carbapenems	Imipenem	100	81.4	**0.008**
Lincosamides	Clindamycin	93.5	89.8	0.063
Polypeptides	Vancomycin	100	100	
Divers	Rifampicin	100	91.5	**0.043**
	Chloramphenicol	63	66.1	0.933

A *p*-value less than 0.05 was considered statistically significant and are in boldface

The difference of MRSA infection was not statistically significant according to gender (*p = 0.42*). Of 59 MRSA strains isolated, 44 (74.6%) were from males while 15 (25.4%) from females. According to category of age, the prevalence of MRSA was 39.0% in ages between 1 and 17 years, 39.3% in ages between 18 and 40 years and 66.7% in ages between 41 and 75 years old. The difference of MRSA distribution was not statistically significant according to age (*p = 0.50*), and health facility (*p = 0.95*).

Specimen-wise distribution showed that MSSA vs MRSA in blood was (44% vs 56%), in ear pus (50% vs 50%), in pus from other sites of the body (44% vs 56%), in urine (33% vs 67%), and in tracheal secretions (50% vs 50%). The specimen wise distribution of MSSA and MRSA was not significantly different (*p = 0.96*).

In males the percentage of MSSA was 31 (41.3%) versus MRSA 44 (58.7%), and in females, MSSA 15 (50%) versus MRSA 15 (50%). The difference of MRSA distribution was not significant according to gender (*p = 0.52*).

### Multi-drug resistance (MDR) pattern of *S. aureus*

Eighty-eight (83.8%) of the isolates were multi-drug resistant. Multi-drug resistant strains ranged from resistance to three classes of antibiotics (11, 10.48%) to 9 classes of antibiotics (1, 0.95%). The highest rate of MDR were observed for 4–5 classes of antibiotics (28, 26.67%). Details of resistance to different antibiotics are described in Table 3.

**Table 3:** Percentage of resistance patterns of *S. aureus* isolates to different number of antibiotics

Antibiotic	Resistant strains	
	No of *S. aureus*	%
P	2	1.91
P,CIP	1	6.67
P,CAZ	3	
P,CFM	2	
P,TOB	1	
P, CAZ, CFM	7	10.48
P, CAZ, CIP	1	
P,E,ATH	1	
P,C,CFM	1	
P,CAZ,SXT	1	
P,CAZ,CFM,DO	1	4.76
P,CAZ,E,ATH	1	
P,TOB,E,ATH	1	
P,CIP,E,ATH	1	
P,CIP,CAZ,CFM	1	
P,TOB,CFM,E,ATH	1	12.38
P,DO,C,CAZ,CFM	2	
P,CAZ,CFM,E,ATH	2	
P,SXT,DO,CAZ,CFM	2	
P,CAZ,OX,FOX,CFM	2	
P,CIP,CAZ,,E,ATH	1	
P,CAZ,OX,FOX,AMC	1	
P,TOB,OX,FOX,AMC	1	
P,DO,CFM,E,ATH	1	
P,CIP,CAZ,CFM,E,ATH	3	8.57
P,CRO,CAZ,OX,FOX,CFM	2	
P,TOB,CAZ,CFM,E,ATH	1	
P,CIP,CRO,CFM,E,ATH	1	
P,SXT,DO,CIP,CAZ,CFM	1	
P,CIP,CAZ,OX,FOX,CFM	1	
P,CRO,CAZ,OX,FOX,CFM,AMC	2	7.62
P,SXT,TOB,CAZ,CFM,E,ATH	1	
P,TOB,DO,CAZ,CFM,E,ATH	1	
P,CIP,CRO,CAZ,CFM,E,ATH	1	
P,SXT,CAZ,CFM,CZ,E,ATH	1	
P,SXT,TOB,G,CIP,CAZ,CFM	1	
P,CAZ,OX,FOX,CFM,E,ATH	1	
P,CIP,CAZ,OX,FOX,CFM,E,ATH	8	13.33
P,SXT,CAZ,OX,FOX,CFM,E,AT	2	
P,RP,C,CAZ,OX,FOX,CFM,CZ	1	
P,CAZ,OX,FOX,CFM,E,ATH,AMC	1	
P,SXT,TOB,CIP,CAZ,OX,FOX,CFM	1	
P,SXT,TOB,CRO,CAZ,OX,FOX,CFM	1	
P,CIP,CRO,CAZ,OX,FOX,CFM,E,ATH	2	13.33
P,CIP,OX,FOX,CFM,CZ,E,ATH,AMC	1	
P,SXT,CIP,CAZ,OX,FOX,CFM,E,ATH	3	
P,SXT,TOB,CAZ,OX,FOX,CFM,E,ATH	1	
P,SXT,CIP,CRO,CAZ,CFM,E,ATH,AMC	1	
P,SXT,TOB,C,CIP,CAZ,OX,FOX,CFM	1	
P,SXT,RP,CAZ,OX,FOX,CFM,E,ATH	1	
P,DO,CIP,CAZ,OX,FOX,CFM,E,ATH	1	
P,SXT,CRO,CAZ,OX,FOX,CFM,E,ATH	3	
P,E,ATH,CFM,FOX,OX,CAZ,CIP,G,TOB	1	1.91
P,AMC,E,ATH,CFM,FOX,OX,CAZ,CIP,DO	1	
P,E,ATH,AMC,CFM,OX,FOX,CAZ,CIP,G,TOB	1	4.76
P,E,ATH,CFM,OX,FOX,CAZ,CIP,DO,G,SXT	1	
P,AMC,CZ,CFM,FOX,OX,CAZ,CRO,CIP,SXT,TOB	1	
P,E,ATH,CFM,FOX,OX,CIP,CD,C,SXT,TOB	1	
P,E,ATH,CZ,CFM,FOX,OX,CAZ,CRO,CIP,TOB	1	
P,E,ATH,AMC,CFM,FOX,OX,CAZ,CRO,G,TOB,SXT	1	1.91
P,AMC,CZ,CFM,FOX,OX,CAZ,CRO,CIP,G,RP,TOB	1	
P,AMC,E,ATH,CFM,FOX,OX,CAZ,CRO,CIP,DO,RP,SXT	1	1.91
P,AMC,E,ATH,CZ,CFM,FOX,OX,CAZ,CRO,CIP,G,TOB	1	
P,E,ATH,CFM,FOX,OX,CAZ,CRO,CIP,CD,DO,G,TOB,SXT	1	0.95
P,AMC,E,ATH,CZ,CFM,FOX,OX,CAZ,CRO,CIP,G,IM,TOB,SXT	5	5.71
P,AMC,E,ATH,CZ,CFM,FOX,OX,CAZ,CRO,CIP,RP,IM,TOB,SXT	1	
P,AMC,E,ATH,CZ,CFM,FOX,OX,CAZ,CRO,CIP,DO,G,IM,TOB,SXT	1	2.86
P,AMC,E,ATH,CZ,CFM,FOX,OX,CAZ,CRO,CIP,CD,G,IM,TOB,SXT	2	
P,AMC,E,ATH,CZ,CFM,FOX,OX,CAZ,CRO,CIP,CD,C,G,IM,TOB,SXT	1	0.95
Total	105	100.0

*P* Penicillin G, *CIP* Ciprofloxacin, *AMC* Amoxicillin-Clavulanic acid, *C* chloramphenicol, *FOX* Cefoxitin, *E* Erythromycin, *ATH* Azithromycin, *G* Gentamicin, *VAN* Vancomycin, *SXT* Trimethoprim-Sulfamethoxazole, *CD* Clindamycin, *CFM* Cefixim, *CZ* Cefazolin, *OX* Oxacillin, *CAZ* Ceftazidime, *CRO* Ceftriaxone, *IM* Imipenem, *TOB* Tobramycin, *RP* Rifampicin, *DO* Doxycycline

## Discussion

In our study, methicillin resistant *S. aureus* was found to be 56.2%. There is no previous information regarding prevalence of MRSA in Afghanistan. In West Asia, MRSA prevalence ranges from 12% to 49.4% in six different hospitals of Saudi Arabia [19]. In European countries, MRSA rates varied from 0.6% in Sweden to 40.2–45% in Belgium, Greece, Ireland, Italy, and the United Kingdom [20, 21], because the use of antibiotics are much more controlled in these countries. In Turkey, the proportion of MRSA were reported to be 50.2% [9] which is similar to European countries. In a study performed in 17 different regions of Russia, methicillin resistance among *S. aureus* strains was between 0% and 89.5% [22] which is very diverse. In a systemic review in Iran, the prevalence of MRSA was determined to be approximately 56.5% (ranged between 50 and 60%) [23], which is similar to our findings and the similarity would be due to irresponsible use of antibiotics in both countries.

We found that the prevalence of MRSA among patients in our study to be 56.2% which is higher compared to findings of a similar study conducted in Peshawar Pakistan, which is very close to Afghanistan. In that, study the researchers examined 280 isolates of *S. aureus* recovered from hospitalized patients, and indicated that 36.1% of *Staphylococci* were detected as MRSA [24]. There was also a significant difference between gender and MRSA infections. In our study, 74% of MRSA isolates were from males. As compared to the study from Pakistan, 34% of MRSA infections were from males. According to age in both studies the prevalence of MRSA infections were higher among elderly in Pakistan and Afghanistan 60.71% and 66.7% respectively, which is a known risk factor for MRSA infections, however in both studies it was not statistically significant*.* The prevalence of MRSA infection in the present study did not vary significantly by gender (*p = 0.42*), age group (*p = 0.50*), specimen (*p = 0.96*) and health facility (*p = 0.95*). This is in agreement with earlier reports by *Geyid* et al. [25] indicating that gender and age are not risk factors for the acquisition or colonization of MRSA.

In our study, despite the high prevalence of MRSA, there was no isolate with reduced susceptibility to vancomycin, however we could not include other new antibiotics like teicoplanin, linezolid and quinupristin/dalfopristin in our study to assess their efficacy as well, because these antibiotics are not included in the licensed and official medicine list of Afghanistan and therefore are not available in Afghanistan [26].

In this study, it was observed that 8.5% of the MRSA strains were resistant to rifampicin and clindamycin and 16.9% were resistant to imipenem; this is probably because these antibiotics are not widely used in the treatment of *Staph* and other bacterial infections in clinics in Afghanistan and are mostly effective in the treatment of sensitive G+ and G- bacteria. Most of the MRSA isolates were resistant to multiple other antimicrobial agents like cefixim (96.6%), ceftazidime (93.2%).

Interestingly, ceftriaxone, which is widely used in Kabul and other provinces of Afghanistan, we found that 44.1% of MRSA strains were resistant to this antibiotic and 49.2% intermediate and only 6.8% were susceptible. This is an alarming sign, which highlights widespread use of this antibiotic and other similar broad spectrum antibiotics in clinical settings and increased resistance toward third generation cephalosporins. In general, elevated rates of multidrug resistance may emerge from diverse isolates of *S. aureus* under antimicrobial pressure or as a result of widespread person to person transmission of multidrug resistant isolates [27]. In our study, although imipenem resistance was detected in 81.4% MRSA strains, no resistance was detected in MSSA strains. In this study, cefazolin, gentamicin and ciprofloxacin were found to be more effective on MSSA than MRSA strains.

Interestingly 8.5% of MRSA strains were resistant to clindamycin, while there was no resistant strain of MSSA to clindamycin. We found that 6.5% of MSSA strains to be intermediate to clindamycin. Our findings support the previous study conducted by Frank, et al. [28] that clindamycin is effective for the treatment of infections caused by *Staphylococci*, or for patients allergic to beta-lactam agents [18, 29]. It is a good alternative to the treatment of both MSSA and MRSA infections.

## Conclusions

The prevalence of MRSA strains obtained in this study was high (56.2%) when compared with the prevalence rates obtained from other similar studies conducted elsewhere. Most of *S. aureus* strains especially MRSA strains were multidrug-resistant and fortunately no isolate was resistant to vancomycin, the drug of choice for treating multidrug resistant MRSA infections. Isolates showed a higher susceptibility to vancomycin, clindamycin, rifampicin, imipenem, amoxicillin + clavulanic acid, cefazolin, gentamicin, and doxycycline. The least effective were azithromycin, ceftriaxone, cefixim and penicillin.

Good infection control practices such as strict hand washing, identifying and treating MRSA carriers, as well as prudent use of antimicrobial agents is recommended. Further, genotypic studies are needed to characterize resistant strains of *S. aureus.*

## Abbreviations

AST: Antibiotic susceptibility testing
ATCC: American Type Culture Collection
CFU: Colony Forming Unit
CLSI: Clinical and Laboratory Standards Institute
FMIC: French Medical Institute for Children
MDR: Multi drug resistant
MRSA: Methicillin resistant *Staphylococcus aureus*
MSSA: Methicillin sensitive *Staphylococcus aureus*
